# An image of portal hypertensive enteropathy in Roux-en-Y gastric bypass anatomy

**DOI:** 10.1093/jcag/gwae009

**Published:** 2024-03-07

**Authors:** Adnan Malik, Shahbaz Qureshi, Abdul Nadir

**Affiliations:** Mountain Vista Medical Center, Mesa, AZ 85209, United States; Mountain Vista Medical Center, Mesa, AZ 85209, United States; Mountain Vista Medical Center, Mesa, AZ 85209, United States

A 60-year-old male with a history of Roux-en-Y gastric bypass, alcohol-related cirrhosis, and oesophageal varices presented with bright red blood per rectum and anemia. Esophagogastroduodenoscopy showed a gastric pouch without varices. Congested, atrophic enteric mucosa and scattered glossy erythematous erosive spots were found adjacent to the gastrojejunostomy anastomosis (Figure 1). Brisk oozing of blood occurred after the jejunal mucosa adjacent to the erosions was biopsied. The bleeding stopped spontaneously after a few minutes of observation. The planned colonoscopy was aborted. He was intubated for airway protection and transferred to the intensive care unit (ICU). Pathology documented jejunal mucosa with focal active inflammation and congested capillaries (Figure 2). A computed tomography (CT) scan of the abdomen showed gastric and splenic varices and portal hypertensive colopathy. He underwent coil embolization of the proximal large and tortuous gastro-renal shunt and an attempted coil-assisted retrograde transvenous obliteration (Figure 3) with cessation of bleeding. However, the patient passed away due to sepsis.

**Figure 1.**
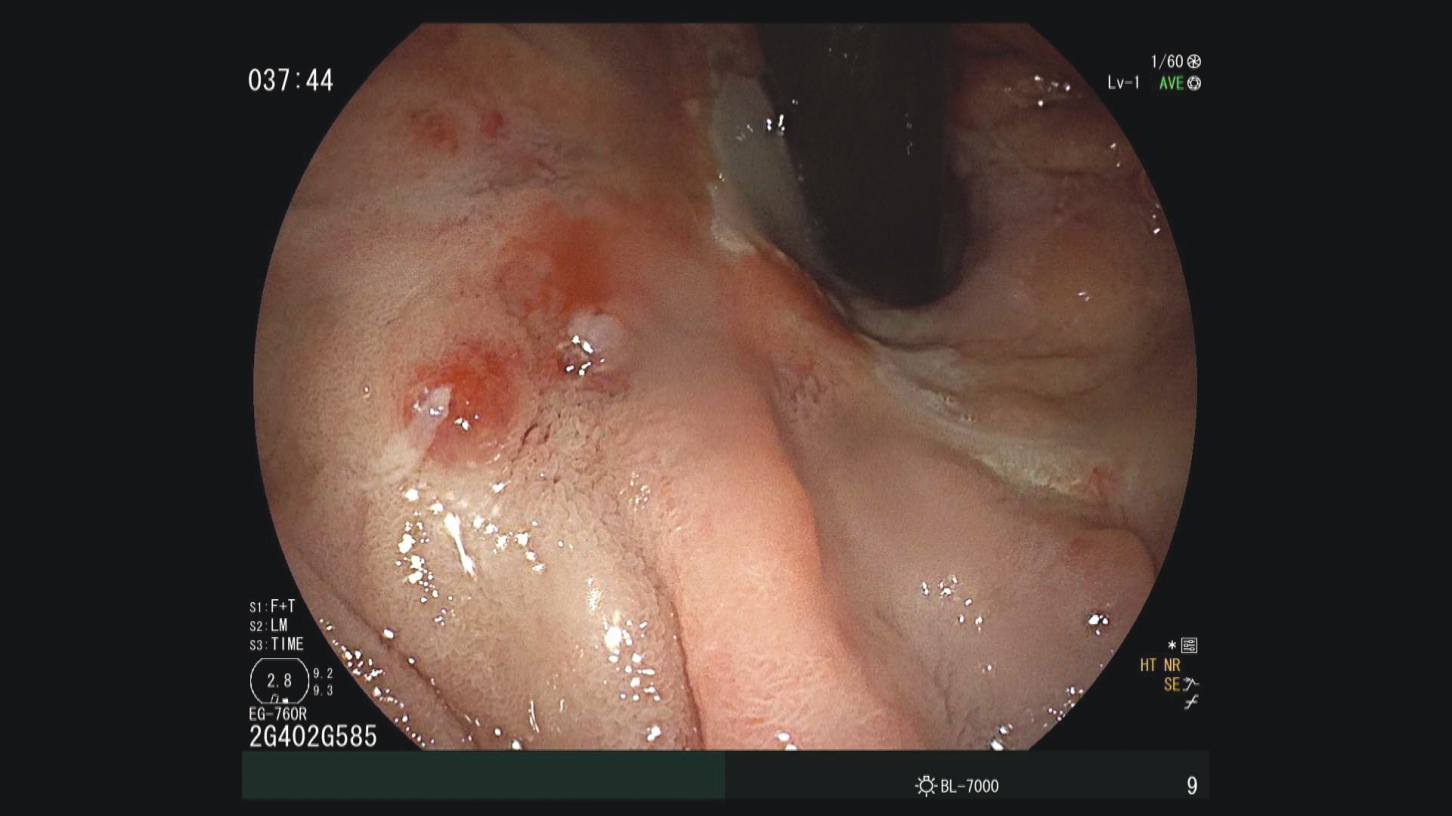
Scattered glossy erythematous and erosive spots at the gastrojejunostomy anastomosis.

**Figure 2.**
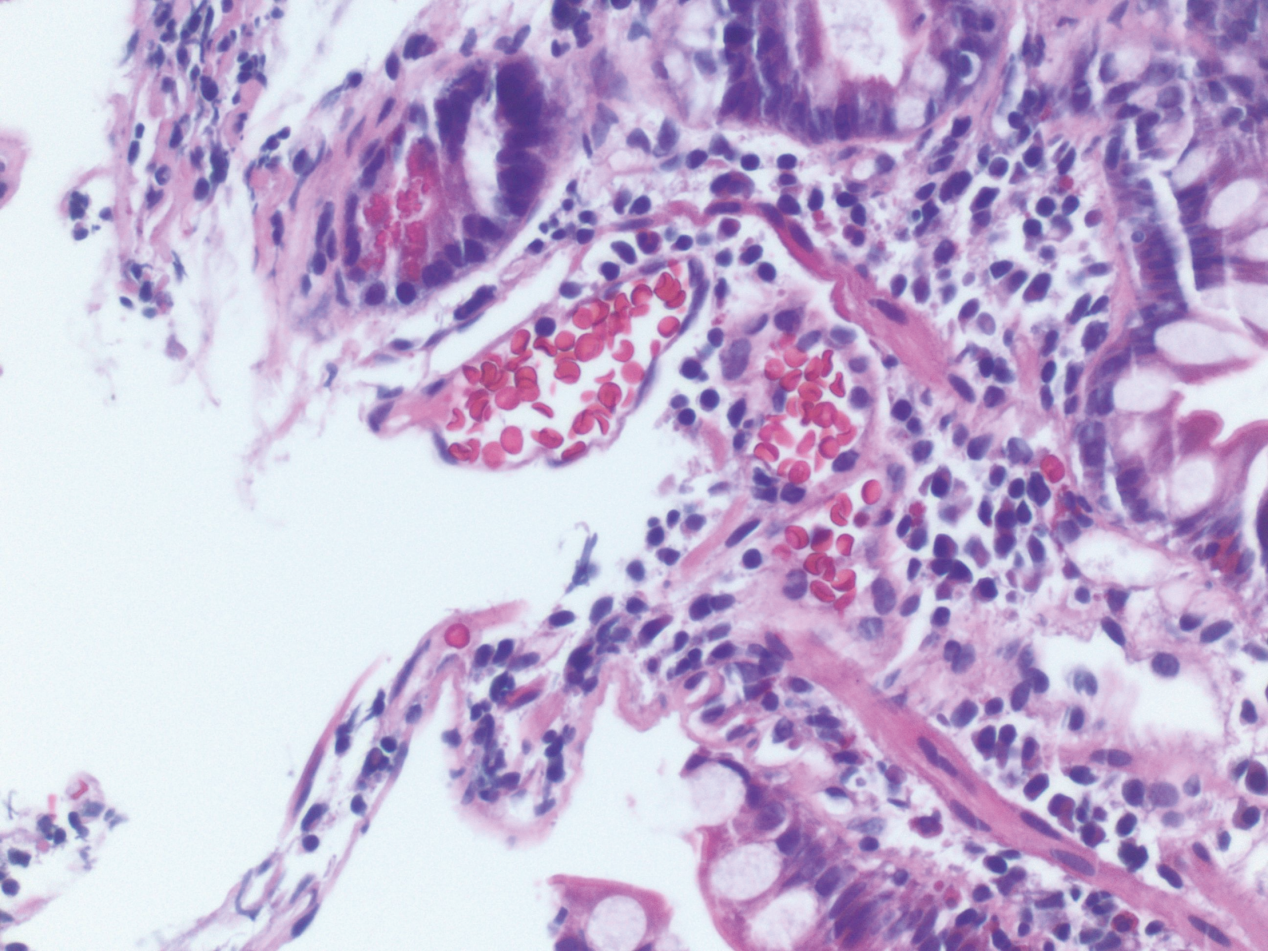
Congested submucosal capillaries in Jejunum-Portal Hypertensive Enteropathy.

**Figure 3.**
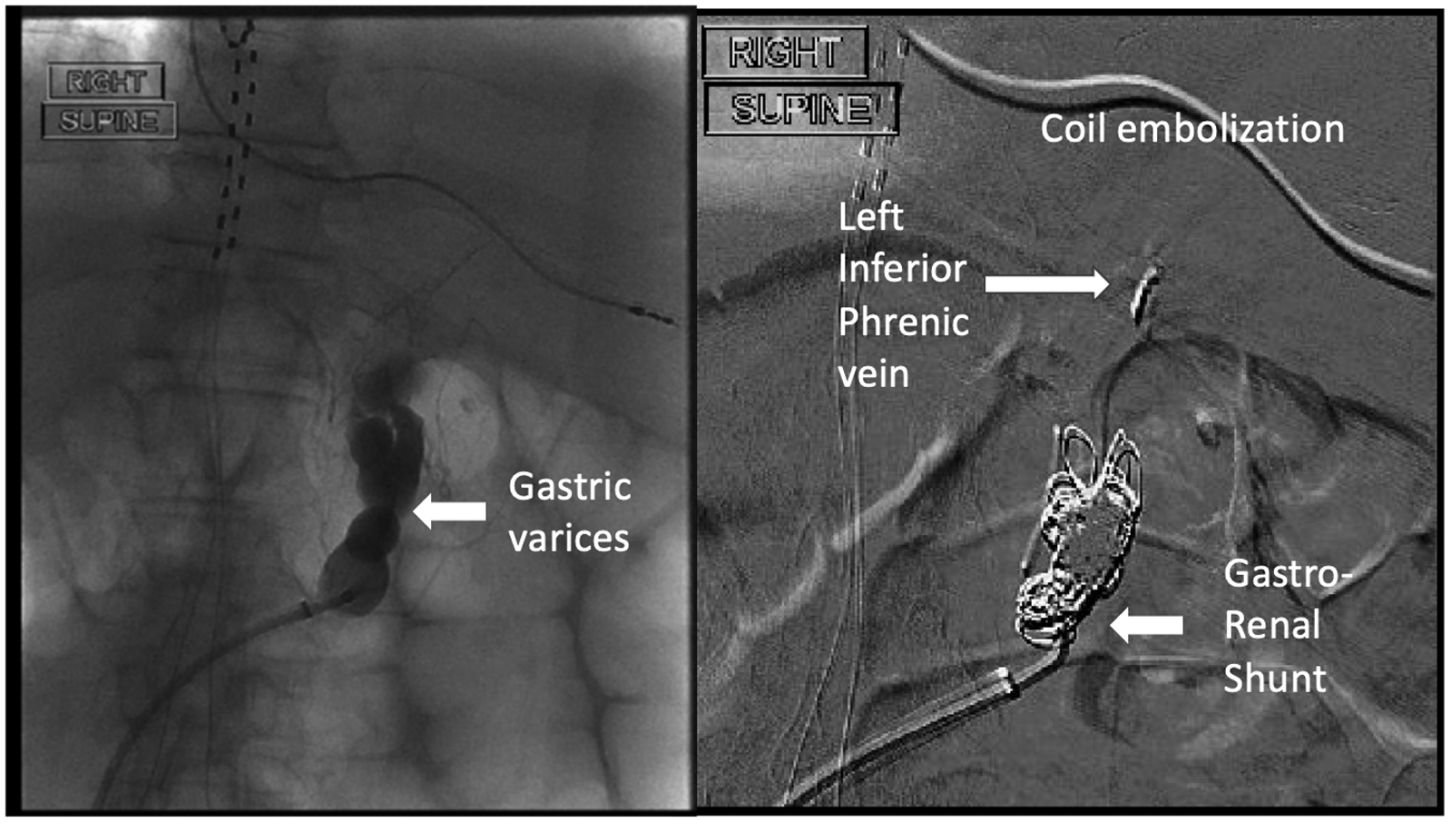
Coil embolization of Gastro-renal Shunt and coil-assisted retrograde transvenous obliteration.

Active alcohol use results in mucosal injury, worsening of portal hypertension, and gastrointestinal (GI) bleeding.^1^ The diagnosis of portal hypertensive enteropathy (PHE) is based on documentation of typical endoscopic and clinical features.^2^ Inflammatory mucosal changes (congestion, villous atrophy, polyps, erythema, friability, and granularity) and vascular lesions (cherry red spots, varices, and angio-dysplastic-like lesions) in the background of portal hypertension conclude the diagnosis of PHE.^3,4^ Red spots, angiodysplasia-like lesions, and active GI bleeding have been reported in 30%–60% and 18% of cases of PHE, respectively.^2^

## Supplementary material

Supplementary material is available online in the Journal of the Canadian Association of Gastroenterology.

gwae009_suppl_Supplementary_ICMJE

## Data Availability

None declared.
